# Supplementary material for: Probe Capture Enrichment Sequencing of amoA Genes Improves the Detection of Diverse Ammonia‐Oxidising Archaeal and Bacterial Populations
Source: Mol Ecol Resour. 2024 Nov 18;25(3):e14042. doi: 10.1111/1755-0998.14042 (PMC11887609; doi:10.1111/1755-0998.14042)
Supplement: Supplementary file 1 — Figures S1 [file MEN-25-e14042-s003.pdf]

Supporting Information

Probe capture enrichment sequencing of *amoA* genes improves the detection of diverse ammonia-oxidizing archaeal and bacterial populations

Satoshi Hiraoka[1†]*, Minoru Ijichi[2†], Hirohiko Takeshima[2], Yohei Kumagai[2], Ching-Chia Yang[2], Yoko Makabe-Kobayashi[2], Hideki Fukuda[2], Susumu Yoshizawa[2], Wataru Iwasaki[2,3], Kazuhiro Kogure[2], Takuhei Shiozaki[2]*

*Correspondence should be addressed to:

hiraokas@jamstec.go.jp, shiozaki@g.ecc.u-tokyo.ac.jp

This PDF file includes:

- Supplementary Tables S1 to S5.
- Supplementary Figures S1 to S6.

Supplementary Tables

Table S1. Summary of leading studies on the distribution of ammonia oxidizers in the ocean.

Table S2. Descriptions of sampling sites and geochemistry.

Table S3. Descriptions and statistics of sequenced reads.

Table S4. RPKMS abundance in metatranscriptomic seawater samples.

Table S5. Mapping ratio of sequencing reads to sequence data at each processing stage.

Supplementary Figures

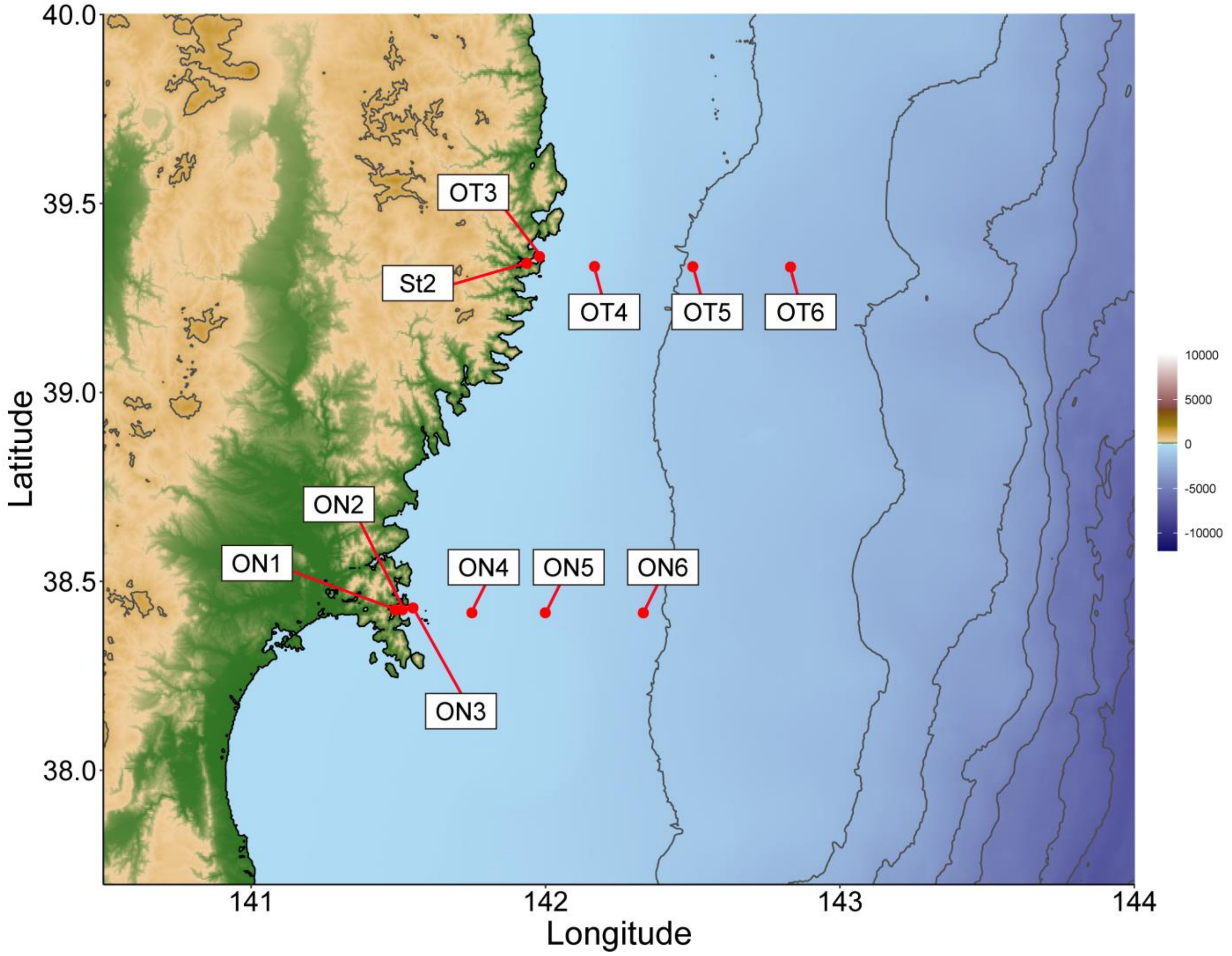

Figure S1. Map of sampling stations along the two transect lines.

The north and south lines are referred to as the Otsuchi (OT) and Onagawa (ON) transects, respectively.

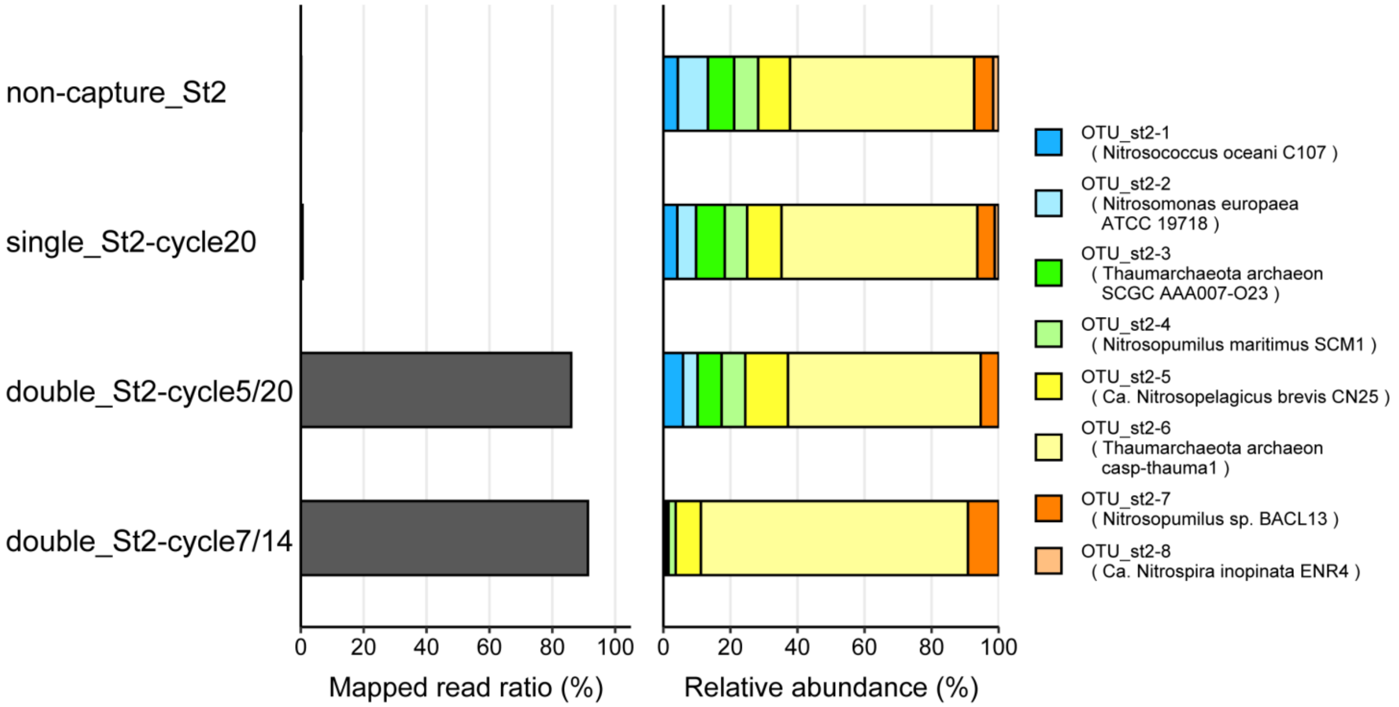

Figure S2. Analysis of metatranscriptomic seawater samples with different hybridization capture and post-capture LM-PCR settings.

The sample was collected at the 0 mbsl layer from the coastal St2 station (St2-0m). See Figure 2.

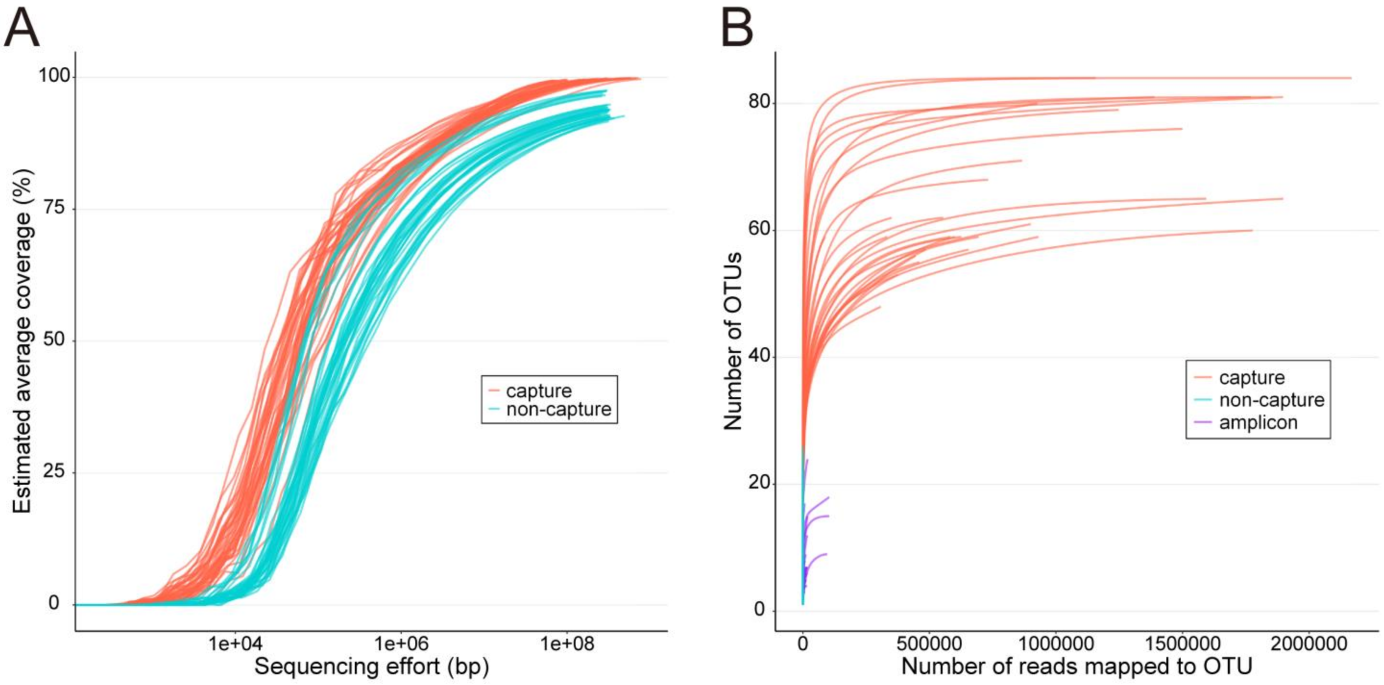

Figure S3. Estimated coverage and rarefaction curve of metatranscriptomic marine samples.

(A) Nonpareil curves of the metatranscriptomic data. Capture and non-capture samples are colored red and blue, respectively. (B) Rarefaction curve of OTU numbers against each of the examined sequencing data. Data from capture, non-capture, and amplicon sequencing settings are colored red, blue, and purple, respectively. Data with less than 100 reads mapped to the OTUs were excluded in this analysis.

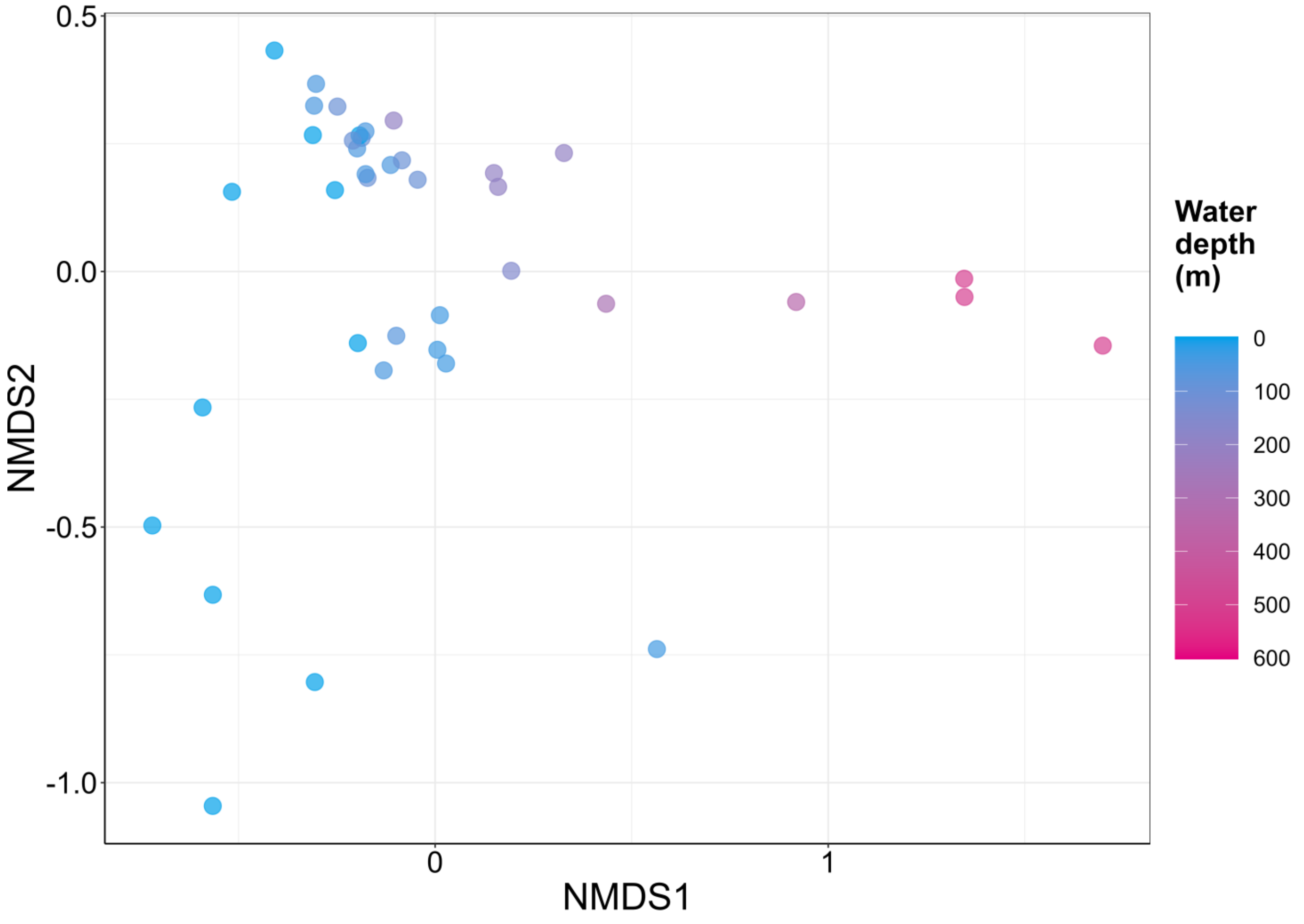

Figure S4. NMDS analysis of RPKMS abundance from metatranscriptomic seawater capture samples.

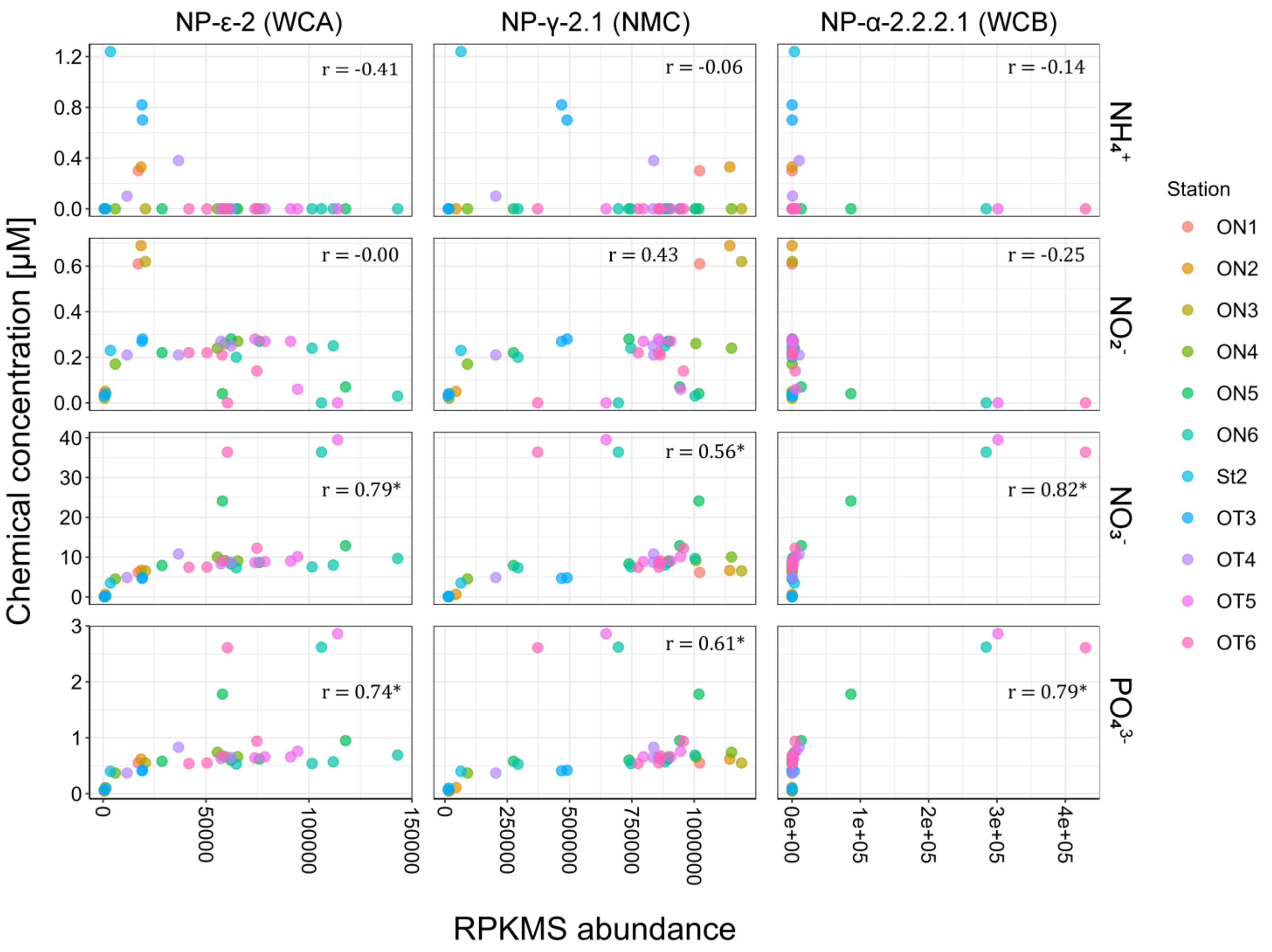

Figure S5. Relationship between the RPKMS abundances of the three ecotypes and seawater chemistry.

Total RPKMS abundances of each ecotype in probe capture enrichment samples were used in this analysis. Each station is coded by color. Spearman's correlation coefficients (r) are shown, with asterisks indicating significant correlations after Bonferroni correction ($q < 0.05$).

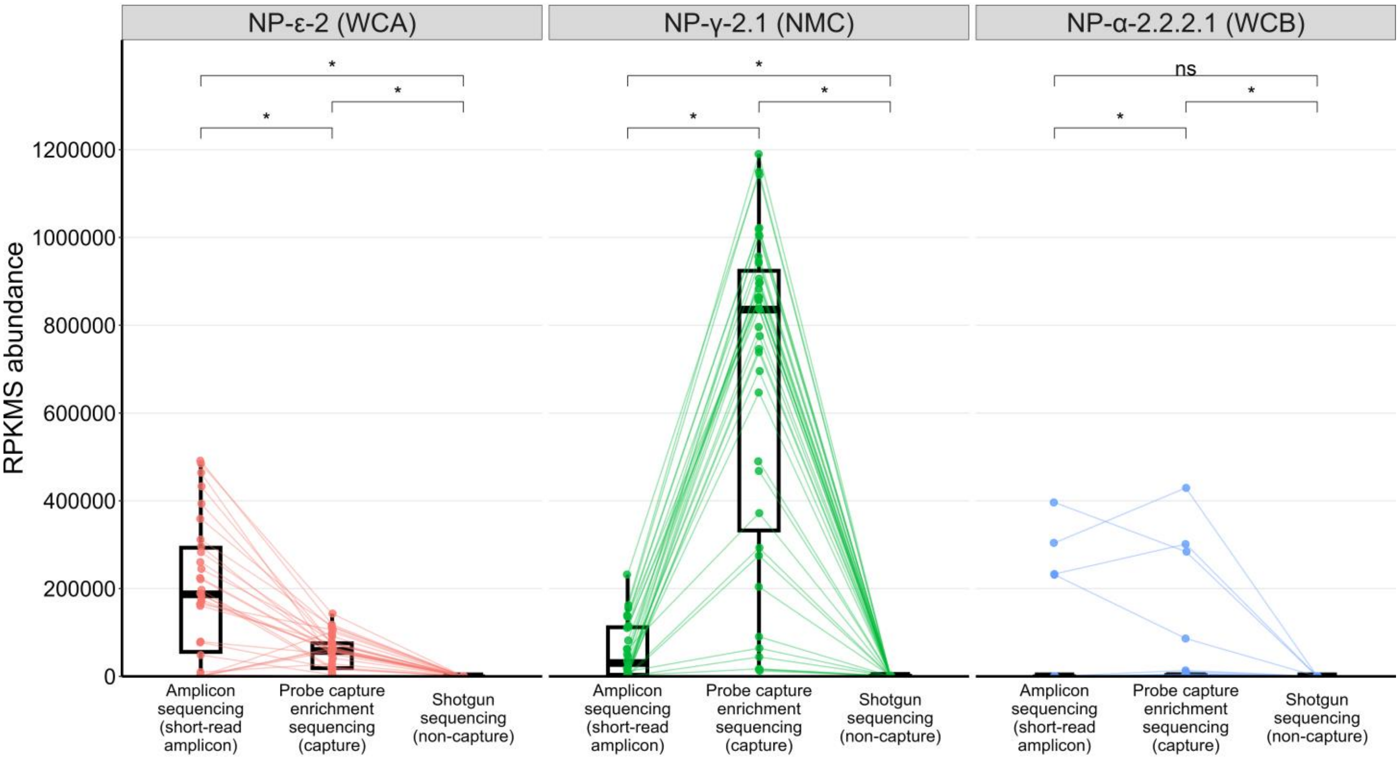

Figure S6. RPKMS abundance of the three ecotypes in amplicon, probe capture enrichment, and shotgun sequencing samples.

Total RPKMS abundances of each ecotype were used in this analysis. Each point represents one sample. Paired samples from the same seawater sample are linked with a line. Asterisk represents statistically significant difference between the sequencing approaches ($p < 0.05$, U-test, Bonferroni correction), while 'ns' represents not significant. Numbers of sample used for the analysis were as follow: N=30 for amplicon sequencing (short-read amplicon), N=39 for probe capture enrichment sequencing (capture), and N=39 for shotgun sequencing (non-capture).
